# Sternalis muscle: an underestimated anterior chest wall anatomical variant

**DOI:** 10.1186/1749-8090-6-73

**Published:** 2011-05-16

**Authors:** Athanasios Raikos, George K Paraskevas, Maria Tzika, Pedro Faustmann, Stefanos Triaridis, Panagiota Kordali, Panagiotis Kitsoulis, Beate Brand-Saberi

**Affiliations:** 1 Department of Anatomy and Molecular Embryology, Medical Faculty, Ruhr University, Bochum, Germany; 2 Department of Anatomy, Medical School, Aristotle University of Thessaloniki, Thessaloniki, Greece; 3 Department of Neuroanatomy and Molecular Brain Research, Medical Faculty, Ruhr University, Bochum, Germany; 4 Department of Otorhinolaryngology Head & Neck Surgery, AHEPA University Hospital, Thessaloniki, Greece

## Abstract

Over the recent years, an increased alertness for thorough knowledge of anatomical variants with clinical significance has been recorded in order to minimize the risks of surgical complications. We report a rare case of bilateral strap-like sternalis muscle of the anterior chest wall in a female cadaver. Its presence may evoke alterations in the electrocardiogram or confuse a routine mammography. The incidental finding of a sternalis muscle in mammography, CT, and MRI studies must be documented in a patient's medical records as it can be used as a pedicle flap or flap microvascular anastomosis during reconstructive surgery of the anterior chest wall, head and neck, and breast. Moreover, its presence may be misdiagnosed as a wide range of benign and malignant anterior chest wall lesions and tumors.

## Background

The sternalis muscle is an anatomical variant of the anterior thoracic region musculature well documented and familiar to anatomists but quite unknown among clinicians and radiologists [1]. It lies superficially and perpendicular to the pectoralis major muscle and parallel to the sternum. Many more terms have been used in the literature to describe sternalis muscle such as "parasternalis" and "rectus sterni" muscle [2,3]. The muscle usually arises from the upper sternum and the infraclavicular region and can display variable insertion points such as the pectoral fascia, lower ribs, costal cartilages, rectus abdominis muscle sheath or the abdominal external oblique muscle aponeurosis [4,5]. However, there is a great variation in height (4.8±1.97 cm), width (15.1±6.84 mm), and thickness (3±0.91 mm) [6]. Its presence ranges from a few short fibers to a well-formed muscle, found unilaterally or bilaterally. Variation also exists in the reported incidence among different populations ranging from 1% in Taiwanese to 18.2% in North Chinese [7,8]. According to Scott-Corner et al, it is more usual in females (8.7%) than in males (6.4%) [9].

The early detection of its presence is critical in regular mammogram screening in order to avoid possible differential diagnostic dilemma. Additionally, there are potential surgical benefits, as it can be used as a flap in reconstruction surgery of the head and neck, anterior chest wall, and breast. We describe a rare observation of a bilateral sternalis muscle in a female cadaver, and we discuss about the muscle's possible embryological origin, innervation, and clinical significance.

## Case presentation

**Figure 1:**
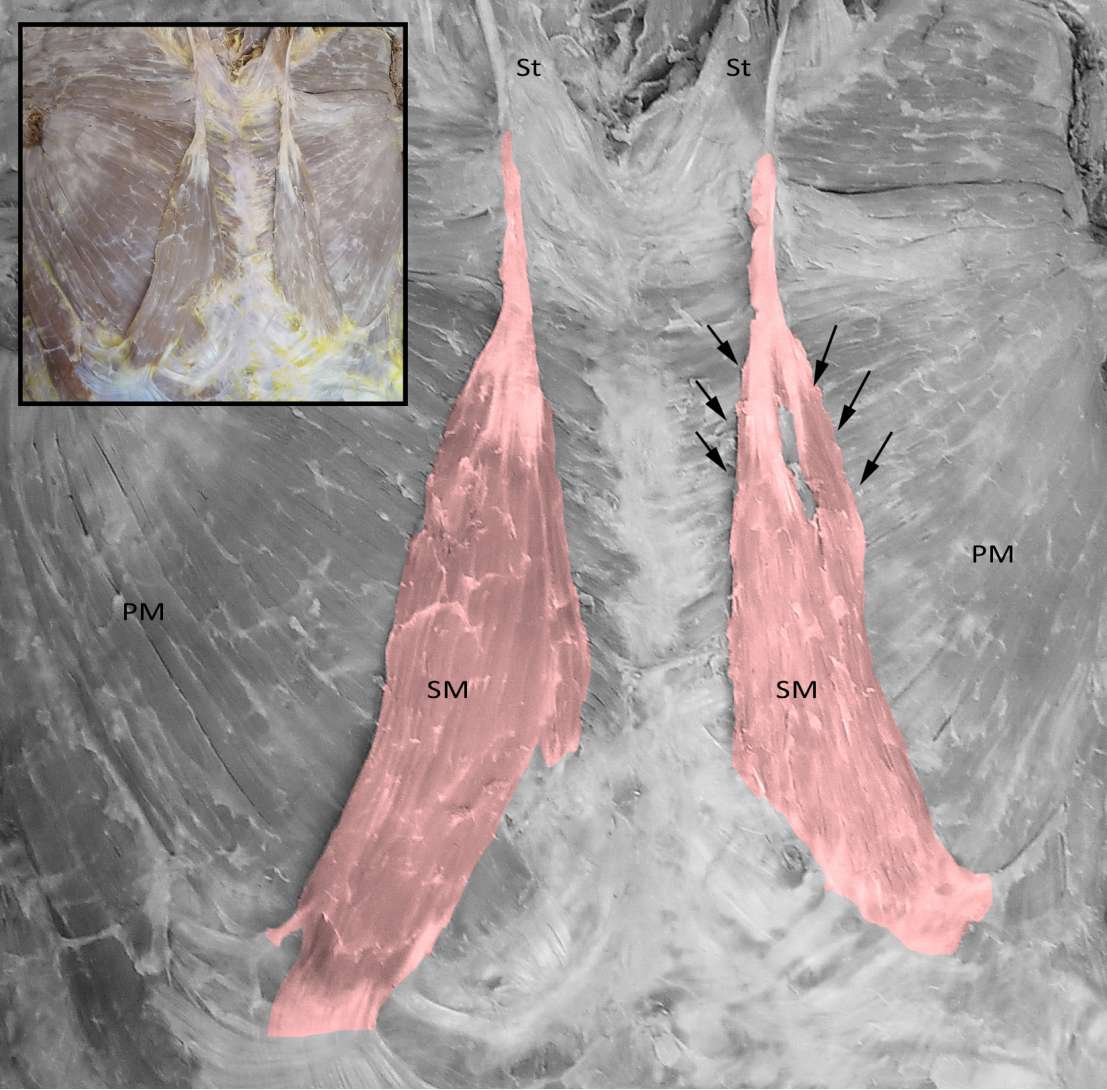
**Bilateral strap-like sternalis muscle (SM) in a female cadaver**. The muscle arises from the tendon of the sternal origin of the sternocleidomastoid muscle (St) and the upper segment of the pectoralis major on each corresponding hemithorax. On the right side, it runs in convex course and terminates at the right sternocostal arch and onto the right external oblique muscle aponeurosis. While on the left, the muscle split into two bellies (arrows) and is inserted onto the left 10^th^-12^th ^costal cartilages and costochondral junctions and terminates on the left sternocostal arch. PM: pectoralis major.

**Figure 2:**
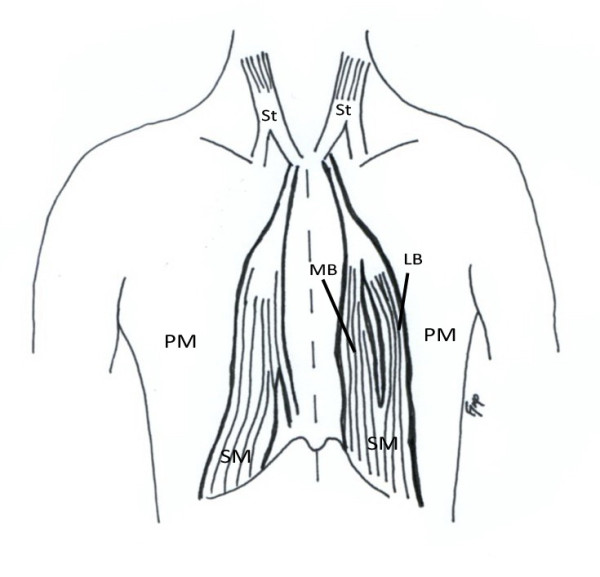
**Schematic representation of Figure 1**. SM: sternalis muscle, St: sternocleidomastoid muscle, PM: pectoralis major. LB: lateral belly, MB: medial belly.

During an educational thoracoabdominal dissection of a female formalin-fixed cadaver, a long well defined vertical muscle was encountered on each hemithorax. Both muscles were strap-like, flattened, located parallel to the sternum in a paramedian position, and lying superficial to the pectoralis major muscle and the pectoral fascia (Figure 1). Specifically, on the right hemithorax, the muscle tendon arose from the sternal origin of the right sternocleidomastoid muscle, and the upper segment of the pectoralis major. The muscle fibers passed downwards in a convex-shaped course and terminated at the right sternocostal arch and onto the right external oblique muscle aponeurosis. On the left side, a bicipital sternalis muscle was observed and although its tendon arose in identical pattern as in the right side, at the level of the sternal angle it split into a medial (wide) and a lateral (narrow) bundle. The muscle gradually inserted onto the left 10^th^-12^th ^costal cartilages and costochondral junctions and terminated on the left sternocostal arch. The schematic representation of the findings can be seen in Figure 2.

According to a classification described by Jelev et al, the bilateral sternalis muscles we describe can be classified as Type I1 and I2 on the right and left side, respectively [10], while according to a recent classification by Raikos et al. it can be classified as Type A on the right and Type C and left side of the thorax [4].

## Discussion

The unilateral sternalis muscle has been reported to be present in 4.5% of subjects, while the bilateral manifestation is found in less than 1.7% [6]. There are many theories to explain the embryological origin of sternalis muscle. It is postulated that it is a derivative of the hypaxial myotomes/dermomyotomes from which the ventral and lateral body wall muscles of thorax and abdomen are developed. Moreover, it is claimed to originate from the adjacent muscles or their blastemas, such as sternocleidomastoid, rectus abdominis, and panniculus carnosus muscle sheet [11], abdominal external oblique muscle or from the ventrolateral part of the diaphragm [4]. Other authors support that the muscle develops either from the rectus abdominis sheath or from pectoralis major due to a defect in the muscle patterning. Particularly, in the latter case, the defective precursor migration of the prepectoral mass which gives rise to the pectoralis major and minor muscles may also contribute to the sternalis muscle development, while mechanical disturbances may lead to atypical clockwise rotation of the muscle fibers [12,13].

Sternalis muscle innervation is quite enigmatic, implying that the rule of the close relationship between muscle ontogeny and innervation may not apply for this muscle. O' Neil and Folan-Curran reported that in 55% of the cases the muscle was innervated from the external or internal thoracic nerves, 43% of the cases it was supplied by the intercostal nerves, while the remaining cases were supplied by both nerves [11]. However, it is quite challenging to preserve the accurate innervation during dissection and surgery because the nerves supplying the muscle may be easily damaged during conventional pectoral fascia dissection, thus a microdissection technique is preferred [12]. Eisler as cited by Schaeffer, found a sternalis muscle in almost half anencephalic fetuses in his investigation, while the frequency in healthy fetuses was significantly lower [14]. A future study focused on individuals or cadavers with neurological history may shed light on the possible connection between sternalis or other muscular variations of the thoracoabdominal region and nervous system. On the other hand, the innervation of muscles depends on local signals, as the growth cones of the axons are known to react to guidance cues [15]. Thus, we propose that the varying innervation of the sternalis muscle is to be expected, and depends on its individual topographical location from where it can attract fibers of different adjacent nerves during development.

The function of sternalis muscle is still unknown. It may participate in the shoulder joint movement or have an accessory role in the lower chest wall elevation [6]. Although its entity has been described for more than 400 years [16], while medical students and physicians are unfamiliar with sternalis muscle variant partially due to insufficient references in standard medical textbooks [5,17,18]. This ignorance may lead to diagnostic dilemma and complications during anterior thoracic region interventions. Other muscles of the anterior chest wall with prominent surgical significance include the transversus thoracis muscle and the axillary arch [19].

The sternalis muscle has never been related to any clinical symptoms. However, its existence may present alterations in the electrocardiogram [2] or confuse a routine mammography [20]. Therefore, it may lead to misdiagnosis, giving the false impression of an irregular structure, often mistaken with a wide range of benign and malignant chest wall and breast lesions, such as breast carcinoma or hematoma (Table 1). Utilization of various imaging techniques, multidetector CT, MRI, and modern techniques such as the 3D volume reconstruction acquired from CT or MRI studies may assist in order to avoid the diagnostic dilemma [4,6]. Sternalis muscle presence may cause breast or chest asymmetry or deviation of the ipsilateral nipple-areola complex [17,21], while it may co-exist with other pectoralis major defects [11,18,22].

**Table 1:** Summary table of sternalis muscle of the anterior thoracic wall.

Etiology	Congenital
**Incidence**	Cadavers: 1-18.2%. Mammograms: 0.019%. Multidetector CT: 6.2%

**Gender ratio**	Slightly higher incidence in females

**Appearance risk factors**	Unknown - Uninvestigated

**Symptoms**	Usually asymptomatic. Minor aesthetic complains and a subject with areola-nipple deviation has been reported so far

**Treatment**	No treatment needed in asymptomatic cases. In reported symptomatic case surgical removal or release was recommended

**Findings on imaging**	Mammogram: Irregular structure medially on the craniocaudal projection. Plain X-ray: Not visible. CT/MRI: Flat and parallel to the sternum structure overlying pectoralis major muscle. 3D reconstruction from CT or MRI: Very helpful in cases of narrow strip-like sternalis muscle

**Differential diagnosis**	Alterations in electrocardiogram. In imaging: may mimic breast carcinoma, fat necrosis, abscess, diabetic mastopathy, hematoma, lymphadenitis, surgical scar, extra-abdominal desmoids tumor, medial insertion of the pectoralis muscle, granular cell tumor, sclerosing adenositis

**Points of surgical interest**	Can be used as an individual or conjoined sternalis-pectoralis muscle flap for breast reconstruction after mastectomy, as a flap in the reconstruction of the anterior chest wall and head & neck

The sternalis muscle can easily confuse the inexperienced because it mimic a variety of benign and malicious conditions [23]. Significant caution should be paid during mammogram interpretation due to a potential blind spot on the median side of the mammography. Craniocaudal projection with adequate traction of the breast may facilitate in inclusion of obscure portions [18]. The awareness of the aberrant structures is important in anterior thoracic wall surgical dissection and particularly in breast, and cardiothoracic surgery. Especially in augmentation mammoplasty, the muscle may interfere with the submuscular pocket dissection causing a different outcome than the expected, whereas due to its insignificant functional importance, sternalis can be used as a muscle flap in anterior chest wall, head and neck, and breast reconstruction [17,24].

## Conclusion

The sternalis muscle is a muscular variant of the anterior chest wall, with an uncertain teleology, function and origin, well known to anatomists but quite unknown to clinicians. The trait must be familiar to anyone intervening in the region in order to avoid diagnostic dilemma or surgical malpractice such as unnecessary mastectomy [10]. In case of diagnostic dilemma, CT and MRI studies may appear helpful.

## Competing interests

The authors declare that they have no competing interests.

## Authors' contributions

AR, GKP, MT, and PK carried out the study design, data analysis and writing, GKP provided the schematic drawing, PF had performed data collection and cadaver dissection. GKP, ST, PKi, and BBS made a critical review of the manuscript. All authors have read and approved the final manuscript.

## References

[B1] Bailey PM Tzarnas CD The sternalis muscle: a normal finding encountered during breast surgery Plast Reconstr Surg 1999 103 1189 1190 10.1097/00006534-199904040-00013 10088505

[B2] Arraez-Aybar LA Sobrado-Perez J Merida-Velasco JR Left musculus sternalis Clin Anat 2003 16 350 354 10.1002/ca.10120 12794922

[B3] Loukas M Bowers M Hullett J Sternalis muscle: a mystery still Folia Morphol (Warsz) 2004 63 147 149 15232768

[B4] Raikos A Paraskevas GK Yusuf F Kordali G Ioannidis O Brand-Saberi B Sternalis muscle A new crossed subtype, classification, and surgical applications Ann Plast Surg 2011 in press 10.1097/SAP.0b013e31820d688b 21407048

[B5] Georgiev GP Jelev L Ovtscharoff VA On the clinical significance of the sternalis muscle Folia Med (Plovdiv) 2009 51 53 56 19957564

[B6] Young Lee B Young Byun J Hee Kim H The sternalis muscles: incidence and imaging findings on MDCT J Thorac Imaging 2006 21 179 183 10.1097/01.rti.0000208287.04490.db 16915061

[B7] Jeng H Su SJ The sternalis muscle: an uncommon anatomical variant among Taiwanese J Anat 1998 193 287 288 10.1046/j.1469-7580.1998.19320287.x 9827644 PMC1467848

[B8] Fukuyama U Der musculus sternalis bei den nordchinesen Okajimas Folia Anat Jpn 1940 19 69 72

[B9] Scott-Conner CE Al-Jurf AS The sternalis muscle Clin Anat 2002 15 67 69 10.1002/ca.1096 11835549

[B10] Jelev L Georgiev G Surchev L The sternalis muscle in the Bulgarian population: classification of sternalis J Anat 2001 199 359 363 10.1046/j.1469-7580.2001.19930359.x 11554516 PMC1468341

[B11] O'Neill MN Folan-Curran J Case report: bilateral sternalis muscles with a bilateral pectoralis major anomaly J Anat 1998 193 289 292 10.1046/j.1469-7580.1998.19320289.x 9827645 PMC1467849

[B12] Kida MY Izumi A Tanaka S Sternalis muscle: topic for debate Clin Anat 2000 13 138 140 10.1002/(SICI)1098-2353(2000)13:2<138::AID-CA8>3.0.CO;2-4 10679858

[B13] Paraskevas GK Raikos A Bilateral pectoral musculature malformations with concomitant vascular anomaly Folia Morphol (Warsz) 2010 69 187 91 21154292

[B14] Schaeffer JP Morris' Human Anatomy 1942 10 Philadelphia: The Blackiston Company 43721604344

[B15] Tessier-Lavigne M Goodman CS The molecular biology of axon guidance Science 1996 274 1123 1133 10.1126/science.274.5290.1123 8895455

[B16] Turner WM On the musculus sternalis J Anat Physiol 1867 1 246 253 17230716 PMC1318550

[B17] Bailey PM Tzarnas CD The sternalis muscle: a normal finding encountered during breast surgery Plast Reconstr Surg 1999 103 1189 1190 10.1097/00006534-199904040-00013 10088505

[B18] Kumar H Rath G Sharma M Kohli M Rani B Bilateral sternalis with unusual left sided presentation: a clinical perspective Yonsei Med J 2003 44 719 722 12950131 10.3349/ymj.2003.44.4.719

[B19] Jelev L Hristov S Ovtscharoff W Variety of transversus thoracis muscle in relation to the internal thoracic artery: a autopsy study of 120 subjects J Cardiothorac Surg 2011 6 11 10.1186/1749-8090-6-11 21272314 PMC3037302

[B20] Brandley FM Hoover HC Jr Hulka CA The sternalis muscle: an unusual normal finding seen on mammography Am J Roentgenol 1996 166 33 66 10.2214/ajr.166.1.8571900 8571900

[B21] Khan UD Use of the rectus sternalis in augmentation mammoplasty: case report and literature search Aesthetic Plast Surg 2008 32 21 24 10.1007/s00266-007-9046-1 17965818

[B22] Kale SS Herrmann G Kalimuthu R Sternomastalis: a variant of the sternalis Ann Plast Surg 2006 56 340 341 10.1097/01.sap.0000197567.42535.d9 16508370

[B23] Kitamura S Yoshioka T Kaneda M A case of the congenital partial defect of the pectoralis major - accompanied by the sternalis with enormous size [in Japanese] Kaibogaku Zasshi 1985 60 728 732 3834728

[B24] Pojchamarnwiputh S Muttarak M Na-Chiangmai W Benign breast lesions mimicking carcinoma at mammography Singapore Med J 2007 48 958 968 17909685

